# How limited cognitive resources impact the attentional effects of self-talk: An eye-tracking study in dart

**DOI:** 10.1371/journal.pone.0319601

**Published:** 2025-03-20

**Authors:** Lu Geng, , Rong Zou, , Jinkun Li, Lin Yu, Xiaobin Hong

**Affiliations:** 1 Department of Psychology, Wuhan Sports University, Wuhan, China; 2 Hubei Key Laboratory of Exercise Training and Monitoring, College of Sports Medicine, Wuhan Sports University, Wuhan, China; 3 Neurocognition and Action - Biomechanics Research Group, Faculty of Psychology and Sports Science, Bielefeld University, Bielefeld, Germany; Morsani College of Medicine, University of South Florida, UNITED STATES OF AMERICA

## Abstract

The study aimed to investigate whether self-talk could enhance participants’ motor performance and attention, even in the presence of distracting interferences and while experiencing ego depletion. To achieve this, 43 novices were randomly assigned into a self-talk group and a control group. Only the self-talk group received the self-talk intervention, and all participants performed a dart throwing task after experiencing ego depletion. In addition, noise interference was added during the dart throwing process. The results indicated that, as predicted, participants in the self-talk group showed enhanced attention functions and more stable motor performance compared to the control group participants, which supports the attentional effect of self-talk. The findings also have practical implications for coaches and athletes. Future research should further use brain science methods to reveal the attentional mechanism by which self-talk improves motor performance when cognitive resources are extremely low and the moderating role of motor experience in this.

## Introduction

It is well known that the sporting environment is often accompanied by different stimuli or interference factors; thus, sports performance demands enough cognitive resources to counter the aversive effects of distraction and ego depletion. “Ego depletion” is a concept that describes a temporary state of reduced resources following the exertion of self-control [1]. According to the Strength Model of Self-Control, self-control is a limited cognitive resource [2,3]. In other words, ego depletion is a phenomenon in which engaging in a self-control task depletes the individual’s self-control resources, leading to a decline in performance during subsequent tasks [4–6]. Self-talk refers to one’s internal dialogue; people use it to analyze events and guide their behavior, whether they express it verbally, internally, or strategically [7].

Athletes’ limited cognitive resources may weaken their performance in a complex sports situation. For instance, ego depletion can make it more challenging to participate in endurance exercises such as push-ups, sit-ups, and power cycling [8–11]. Moreover, ego depletion impairs performance in fine motor tasks by negatively affecting attentional regulation [12,13]. Fine muscle movements—such as basketball shooting and dart throwing that demand more attentional resources—are more likely to be negatively impacted by ego depletion [12,14]. This may be attributed to athletes’ limited cognitive resources, which are further diminished by ego depletion, rendering them unable to defend themselves against interferences. This highlights the importance of self-control resources in athletic performance [6,15]. Is there any way to alleviate the negative impact of ego depletion on sports performance? Research about the effects of self-talk on athletes’ performance has expanded rapidly since 2006 [16,17]. Self-talk is a valuable psychological tool that can be easily implemented to enhance performance because it can reduce the unpleasant consequences of ego depletion and improve attentional focus and performance [18]. Research in sports psychology has also underscored the significance of self-talk as a powerful tool for motivation, inducing positive psychological states, boosting confidence, improving scores, increasing concentration, and reducing negative thoughts [19–23]. Subsequently, research has begun to explore whether self-talk can improve attention function during ego depletion. Gregersen et al. [24] found that participants in the self-talk group performed better on visual and audio tests while experiencing ego depletion. To increase the ecological validity of self-talk research in sports, studies investigated the effects of a self-talk intervention on golf-putting performance in individuals experiencing ego depletion, and it concluded that self-talk reduced the effects of ego depletion on performance [25] and improved attention function [26] during a golf-putting task, demonstrating its effectiveness as a strategy for enhancing athletic performance. In actual sports, people will often face various stressful situations, resulting in cognitive resource loss that is not conducive to performance. Studies have shown that self-talk can improve athletes’ performance under stress [27], and a recent meta-analysis also showed that psychological training techniques such as self-talk are useful strategies to promote athletes’ performance under stress [28].

Additionally, external distractions may hinder athletes’ performance. According to previous research, self-talk can help mitigate the negative effects of limited cognitive resources and distracting interferences. For instance, a study that combined field and laboratory tests showed that self-talk can improve task performance in the presence of noise interference [29]. Furthermore, employing motivational and instructional self-talk may counter the adverse effects of distraction on performance [30,31]. The limited capacity model of attention assumes that one’s total attentional capacity at any one point in time is restricted [32]; distraction is known to reduce available attentional resources. Self-talk can help preserve or replenish attentional resources, enhance self-control, and facilitate motor performance [18]. As a result, self-talk plays a crucial role in self-control as well as in sports cognition and performance when athletes experience distracted states. This phenomenon, known as the “attentional effect of self-talk” explains why self-talk boosts sports performance by preserving attentional resources [18,29,33]. The study found that self-talk improves attention and improves emotional control and confidence in novice athletes [34]. Recent studies have also found that self-talk can resist noise interference to improve attentional control [35], and also enhance attention to improve task performance [36]. Furthermore, studies have investigated the effect of self-talk on attention by analyzing gaze behaviors. For example, Galanis et al. [37] used pupillometry to examine the effect of self-talk on mental effort; the self-talk group showed smaller pupillary distances and higher scores compared to the control group. This suggests that self-talk reduces mental effort and leads to more relaxed attentional performance. Another study found through mediation analysis that self-talk improved motor performance by extending quiet eye duration [38]. In conclusion, the aforementioned studies confirm the attentional effects of self-talk. However, previous research has only examined the attentional effects of self-talk within the context of a single influencing factor, such as the effect of self-talk on attention during a state of ego depletion or distraction. Given the complex factors that influence motor performance before and during actual competitions, it is important to understand and analyze them thoroughly. If the motor task of resisting distraction is performed after experiencing ego depletion, it is necessary to determine whether self-talk still improves attention and facilitates performance when cognitive resources are extremely depleted. Hence, we aimed to examine the attentional effects of self-talk when cognitive resources are extremely limited.

Diverse studies have covered the interesting topic of the relationship between eye movements and attention. Gaze behaviors provide a window into the attentional system’s functioning [39]. Shepherd et al. [40] found that while it is possible to shift attention without corresponding eye movements, it is not possible to make an eye movement without a corresponding shift in attention. This indicates a strong connection between where attention is focused and where the eyes fixate. Gaze behaviors —which are behavioral indicators of focused attention—have not been previously used to explore the attentional effects of self-talk when cognitive resources are extremely limited. To enhance ecological validity, we randomly assigned novices to two groups. Only the self-talk group received the self-talk intervention, and all participants performed a dart throwing task after experiencing ego depletion. Additionally, noise interference was added during the dart throwing process. Using the mobile eye tracker, we recorded eye movements and investigated the differences in motor performance between the two groups. As the participants were all novices, the experimenter consulted several amateurs in the game of darts in advance to avoid the floor effect. We ultimately decided to divide the areas of interest (AOI) into the following: the “central” zone (throwing more than eight rings) and the “near-central” zone (throwing 1–7 rings). We posited that self-talk would prevent the participants from becoming distracted when completing a motor task during ego depletion, and during this period, self-talk could preserve participants’ cognitive resources and increase their attention and motor performance. Specifically, compared with the control group, the self-talk group exhibited a higher fixation count and visit count in the near-central and central zones of the dartboard, as well as longer fixation duration and visit duration. Additionally, the dart performance of the self-talk group was superior to that of the control group.

## Experiment

### Method

#### Participants.

According to the calculation of G * Power 3.1, the minimum number of participants was 34. In the end, we recruited 43 men (*M* =  18.67, *SD* =  2.33), all of whom were right-handed, had normal vision, and had no prior experience. Following a baseline test, people who were requested to get enough sleep were randomized into two groups: the self-talk group (22 participants) and the control group (21 participants). The research protocol was reviewed and approved by the Medical Ethics Committee of the first author’s university. The participants provided their written informed consent and their parents agreed to participate in the experiment. The start and end dates of the experiment are November 10, 2023 to January 21, 2024.

#### Materials.

(1) Tobii Glasses: For the experiment, we used the Tobii Pro Glasses 2, a wearable eye-tracker with real-time viewing capabilities that was created in Sweden and comes with an adjustable nosepiece and a stabilized device to lessen vibration. The eye-tracker is equipped with four eye-tracking cameras that have a resolution of 1920 ×  1080, a sampling rate of 50 Hz, and a scene camera tracking angles of 82° horizontally and 52° vertically.
(2) A Lenovo G40-70 portable laptop: A Lenovo G40-70 portable laptop was equipped with Tobii Pro Glasses 2 Controller software for data recording and logging, and Tobii Pro Glasses Analyzer software was used for video playback and data visualization and analysis.
(3) A dartboard and five darts: An 18-inch international standard dartboard was used for the experiment (for novices, it is too difficult to use a professional dartboard). The darts were professional ones and made of pure copper. They weighed 25 grams. We also used ten ping-pong balls, several cups, and several coins.
(4) Background noise audio and wireless Bluetooth headset: We created a background noise playback that was sudden, discontinuous (10 seconds on, 5 seconds off), and loud (about 95 dB). This volume is considered sufficiently loud to distract an individual and hinder performance. We wore the headphones in advance to feel the background noise. As 95 dB could have caused hearing damage to the participants, we set the noise stimulus in the experiment to 65 dB (within the noise value range), and employed an abrupt, discontinuous cyclic playback pattern (10 seconds on, 5 seconds off).

#### Procedure.

##### Pre-test phase

Before the experiment, all participants went to the laboratory to read the informed consent form and provide basic personal information.

The experiment was conducted in an eye-tracking laboratory without an infrared light source. The laboratory set was a clean and simple white color to reduce external influences on the participants. The bullseye was set at a height of approximately 1.70 m above the floor, and the dartboard was set at a distance of approximately 2.30 m from the throwing line. All participants were allowed to practice throwing darts for 3 – 5 min to familiarize themselves with the relevant movements. During formal throwing, the participants wore the eye-tracking device to perform 20 throws, and the experimenter recorded their scores. The participants were asked to keep their heads as stable as possible while throwing. As for the scoring rules, the number of rings corresponded to the score one by one: 10 points for hitting the bullseye, 1 point for hitting the outermost ring, and 0 points for hitting off-target.

##### Intervention phase

The self-talk intervention has been found to improve task performance [19]. Additionally, the intervention’s goal is to teach participants how to utilize self-talk. First, the direct use of self-talk to novices may cause them to feel uneasy and distracted, which could potentially diminish the effectiveness of self-talk. Thus, in the experiment, we established a three-day self-talk intervention phase. Second, we selected “throwing a table tennis ball into a cup” as the intervention activity for practicing self-talk to prevent the practice effect from influencing the experiment’s outcomes. The self-talk group was expected to complete the intervention task for about 30 min per day for three days during the intervention phase.

Before starting the intervention, the participants in the self-talk group were given an explanation of self-talk. Then, the participants were informed that the next intervention would involve content on instructional self-talk; for example, they were told, “Please aim at the cup and apply appropriate force” when throwing. A honeycomb hexagon made of several plastic cups in red, yellow, pink, blue, and white was set up, with the central cup situated approximately 2 m away from the throwing line. There was only one red cup in the hexagon’s center; the remaining cups were organized in circles, one after the other. The experimenter pre-positioned some coins in the cups (the red cup in the central having the most) to increase the participants’ motivation. The experimenter asked each participant about using self-talk at the end of each intervention activity and had them provide a verbal response: “How often did you use self-talk during the throwing process?” The participants answered using a 10-point Likert scale where 1 =  “not at all” and 10 =  “utilized it continuously.” The participants orally reported that they used the designated self-talk content throughout the entire process (*M* =  9.32, *SD* =  0.89). The control group participants were not given a self-talk explanation, and they conducted the “throwing a table tennis ball into a cup” as the intervention activity directly.

##### Post-test phase

Before beginning the task, ego depletion transcription material was used to transcribe by both groups of participants. For details, please refer to the pilot test to verify the validity of the ego depletion transcription material in the S1 File. Very little research has looked at the duration of the ego depletion effect, and the findings that do exist are inconsistent. According to Gregersen et al.’s [24] experimental manipulation, the period between the ego depletion task and the dart task in the current study was roughly 3 min, during which time all experimental preparations were made. The dart throwing rules of the post-test stage were identical to those of the pre-test stage.

#### Measures.

At the end, the participants filled out the Self-Talk Manipulation Check questionnaire and the College Student Self-Control Scale. The process of self-talk manipulation check referenced methods used in previous research [41,42]. After the throwing task, the participants filled out the Self-Talk Manipulation Check questionnaire, which involved a 10-point Likert scale. The self-talk group was asked the following questions:

(1) Did other unrelated thoughts affect the way you performed the task?
(2) Before each act of throwing, did you use instructional self-talk?
(3) If you used self-talk, to what extent did you use it?
(4) Do you remember the content of the self-talk? Please write down what you remember.
(5) Did you use other forms of self-talk throughout the task? If not, write “No”. If you did, please write down what the content was and the extent to which you used it.

The control group was asked the following questions:

(1) Did other unrelated thoughts affect the way you performed the task?
(2) Did you use any form of self-talk throughout the task? If no, please write “None”. If yes, please write down what you used and to what extent.

A revised Chinese version of the College Student Self-Control Scale developed by Tan and Guo [43] was used. The scale contains 19 items and has good reliability and validity, with an internal consistency reliability of 0.862 and a re-test reliability of 0.850. The internal consistency coefficient α =  0.839 was measured in the current experiment. People with different traits of self-control have different self-control abilities [44,45]. To exclude between-group differences in trait self-control, we administered the College Student Self-Control Scale to all participants at the end of the experiment.

#### Data recording and analysis.

We collected the gaze behaviors using the Tobii Pro Glasses 2 Controller software. The Tobii Pro Glasses Analyzer program was then utilized for data processing and visualization. We manually matched the fixation points before delineating the areas of interest after playing back the experimental video in the Tobii Pro Glasses Analyzer software. This allowed us to record the time between the experimenter’s command to “start” and the moment the dart was thrown.

Before the experiment began, the participants were asked to wear the mobile eye tracker and identify the fixation point on the wall. The experimenter confirmed the correctness of their fixation point to ensure the quality of data collection and calibration. Based on our goals, the indexes used for the present study were the fixation count, visit count, fixation duration, and visit duration.

The experiment involved a pre-test and a post-test. Due to the dart performance (scores) and the gaze behaviors were non-normal distribution, according to the Shapiro-Wilk test, post-test dart scores and post-test eye tracking index were presented using medians and interquartile ranges (IQR), and a Mann-Whitney test was used to compare dart scores and eye tracking index between groups.

The self-talk group had 22 participants, and one of them used motivational self-talk rather than instructional self-talk as required in the task; we thus eliminated their data. We also eliminated the data of three participants as we had problems matching their data, leaving the self-talk group with 18 valid sets of data. There were 21 participants in the control group, and 4 of them used distinct instructional self-talk on their own, resulting in a total of 17 valid sets of data for the control group.

## Results

### Non-parametric tests of pre-test and post-test dart scores for both groups

First, the non-parametric Mann-Whitney test was used to analyze the pre-test dart scores, and there was no difference between the two groups (*Z* =  -1.52, *p* >  0.05). Then the Mann-Whitney test was used to analyse the post-test dart scores, which showed that the extremely significant difference between the two groups (*Z* =  -4.37, *p* < 0.001, *η*^2^_*p*_ =  0.55), the performance of the self-talk group (median: 103.87; IQR: 3.52) was higher than that of the control group (median: 100.87; IQR: 0.75).

This result confirms the research hypothesis that the self-talk intervention can fully improve the sport performance when the participants are under ego depletion and resisting distraction interference.

### Non-parametric tests of pre-test and post-test eye tracking index for both groups

First, the the Mann-Whitney test was conducted on the pre-test eye tracking index of the two groups, and the results found that the difference was not significant between the two groups. Then the the Mann-Whitney test was conducted on the post-test eye tracking index of the two groups, the results showed that the significant difference of fixation duration in the near-central zone (*Z* =  -2.27, *p* < 0.05, *η*^2^_*p*_ =  0.15), the self-talk group had a significantly longer fixation duration (median: 46.79; IQR: 3.95) than the control group (median: 39.00; IQR: 2.37); the extremely significant difference of fixation duration in the central zone of the two groups (*Z* =  -4.82, *p* <  0.001, *η*^2^_*p*_ =  0.66), the self-talk group had a significantly longer fixation duration (median: 11.00; IQR: 1.62) than the control group (median: 5.70; IQR:1.61); the extremely significant difference of fixation count in the near-central zone of the two groups (*Z* =  -3.66, *p* <  0.001, *η*^2^_*p*_ =  0.38), the self-talk group fixated on more points in the near-central zone to a significant extent (median: 69.17; IQR: 12.61) than the control group (median: 54.00; IQR: 17.50); the extremely significant difference of fixation count in the central zone of the two groups (*Z* =  -4.19, *p* <  0.001, *η*^2^_*p*_ =  0.50), the self-talk group fixated on more points in the central zone to a significant extent (median: 21.90; IQR: 6.84) than the control group (median: 14.00; IQR: 6.50); the effect of the self-talk intervention on the visit duration in the near-central zone was significant(*Z* =  -3.36, *p* <  0.01, *η*^2^_*p*_ =  0.32), the self-talk group had a significantly longer visit duration (median: 54.41; IQR: 19.91) than the control group (median: 44.20; IQR: 21.56); the self-talk intervention had a extremely significant effect on visit duration in the central zone(*Z* =  -4.98, *p* <  0.001, *η*^2^_*p*_ =  0.71), with the participants in the self-talk group having visited the central zone for a significantly longer period(median: 12.14; IQR: 2.55) than the participants in the control group (median: 8.77; IQR: 2.85); but the effect of the self-talk intervention on the visit count in the near-central zone was not significant (*Z* =  -1.28, *p* >  0.05); the significant difference of visit count in the central zone of the two groups (*Z* =  -3.43, *p* <  0.01, *η*^2^_*p*_ =  0.34), the self-talk group fixated on more points in the central zone to a significant extent (median: 10.81; IQR: 5.27) than the control group (median: 8.00; IQR: 1.50). The results are shown in Table 1.

**Table 1:** Description analysis of the study variables.

Variables	Median(IQR)		*p*	*η* ^*2*^
Self-talk group	Control group
**Central zone**				
fixation duration	11.00 (1.62)	5.70 (1.61)	0.000^***^	0.66
visit duration	12.14 (2.55)	8.77 (2.85)	0.000^***^	0.71
fixation count	21.90 (6.84)	14.00 (6.50)	0.000^***^	0.50
visit count	10.81 (5.27)	8.00 (1.50)	0.001^**^	0.34
**Near-central zone**				
fixation duration	46.79 (3.95)	39.00 (2.37)	0.024^*^	0.15
visit duration	54.41 (19.91)	44.20 (21.56)	0.001^**^	0.32
fixation count	69.17 (12.61)	54.00 (17.50)	0.000^***^	0.38
visit count	17.33 (5.75)	16.00 (5.00)	0.200	0.05

* Significant at *p* <  0.05.

** =  *p* <  0.01.

*** = *p* < 0.001.

According to the above results, it can be concluded that the fixation duration and visit duration in the near-central zone and the central zone of the self-talk group were longer than that of the control group, and the fixation count and visit count in the self-talk group were basically more than that of the control group. The results fully confirm the hypothesis, indicating that self-talk intervention can improve the attention of participants when cognitive resources are extremely limited, and the results again verify the attentional effect of self-talk.

To further capture the relationship between gaze behavior and actual performance, the next analysis is to use Spearman’s correlation rho efficient tests to explore the relationship between eye movement and dart performance.

### Correlation test between dart performance and eye tracking index

First, the results are shown that a significant positive relationship between the post-test score and the post-test eye tracking index of the overall participants (*p* <  0.05).

According to the purpose of the study, the study focuses on the correlation between the post-test scores and the post-test eye tracking index in the self-talk group.

Furthermore, a subanalysis of self-talk group also showed a positive correlation between the dart score and the fixation duration in the near-central zone (*p* <  0.01; r =  0.66); in addition, the results were also found that a positive correlation between the dart score and the visit duration in the near-central zone (*p* <  0.001; r =  0.93); a positive correlation between the dart score and the visit count in the near-central zone (*p* <  0.001; r =  0.84); a positive correlation between the dart score and the visit count in the central zone (*p* <  0.01; r =  0.65). The results are shown in Table 2.

**Table 2:** Correlations between dart score and eye tracking index for self-talk group (N =  18).

Variable	Spearman’s correlations (r)	Central zone				Near-central zone			
fixation duration	visit duration	fixation count	visit count	fixation duration	visit duration	fixation count	visit count
**dart score**	r	0.11	0.07	0.06	0.65^**^	0.66^**^	0.93^***^	0.03	0.84^***^
	*P*	0.62	0.75	0.78	0.001	0.001	0.000	0.90	0.000

* Significant at *p* <  0.05.

** =  *p* <  0.01.

*** = *p* < 0.001.

The above results indicate a significant positive correlation between gaze behavior and actual motor performance, which further supports the effectiveness of the self-talk intervention.

### Visualization graphs of the two groups

**Fig 1:**
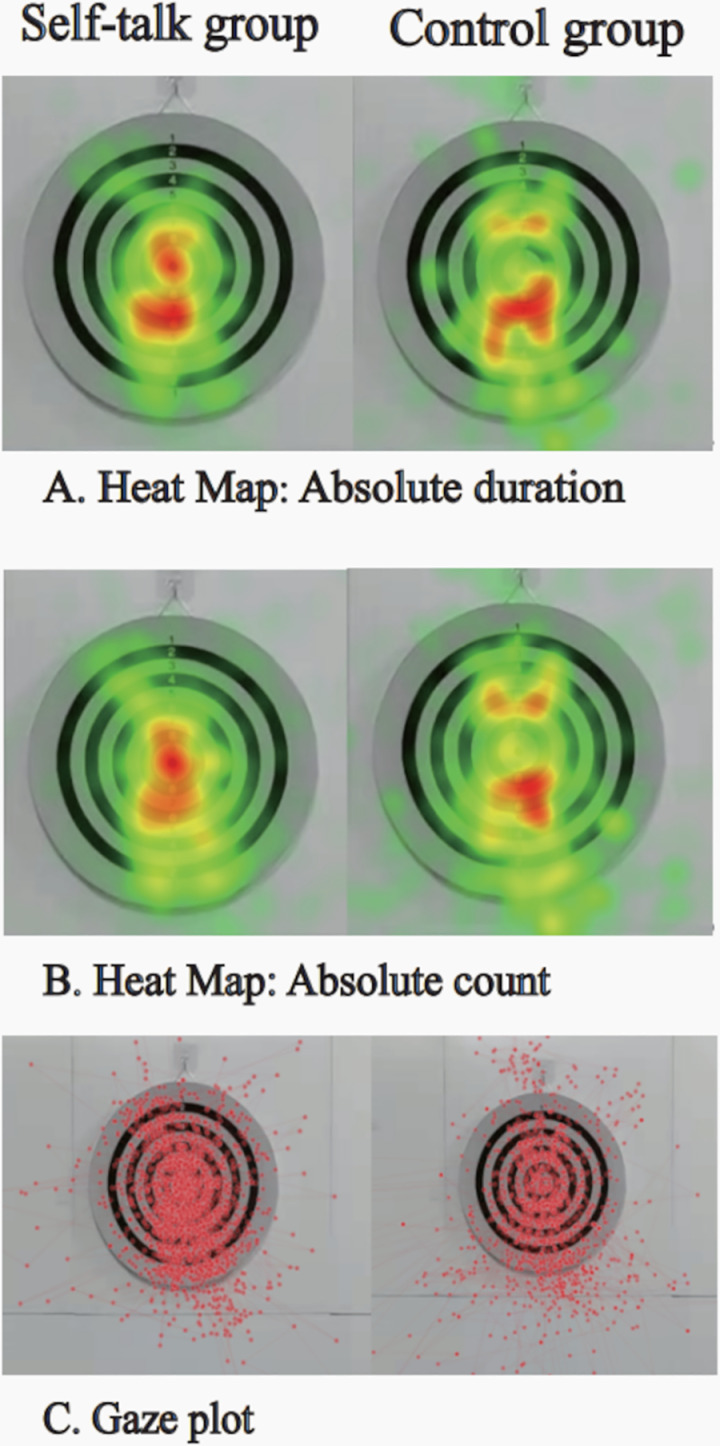
Visualization graphs: Heat maps and gaze plots. (A) shows the fixation duration, with a redder shade representing a longer fixation duration and a greener shade denoting a shorter fixation duration. (B) presents the number of fixations, with a redder shade implying more fixations and a greener shade representing fewer fixations. The red dot in (C) indicates the fixation point, and the connecting line denotes the fixation trajectory.

Fig 1(A) shows the fixation duration heat maps of the two groups; the participants in the self-talk group have more red distributions in the central zone than the participants in the control group, whereas the participants in the control group have a more dispersed distribution of red regions. Fig 1(B) illustrates the heat maps of the fixation count between the two groups; the participants in the self-talk group have a more red distribution in the central zone and are more centrally compact than the participants in the control group. Fig 1(C) presents the gaze plots of the two groups’ gaze behaviors. The gaze trajectories of the self-talk group are concentrated in the dartboard zone; the overall trajectory is more stable, whereas the gaze trajectories of the control group are more dispersed.

## Discussion

Self-talk has been widely recognized as a beneficial psychological technique in sports, with numerous studies supporting its effectiveness in enhancing athletic performance. Self-talk interventions can assist participants in resisting external distractions and subsequently improving attention function. Self-talk can also facilitate more stable performance. Based on existing research, the current study explored the possibility that self-talk could still enhance attention and athletic performance when people resisted distractions while playing darts in a state of ego depletion. We used visit duration, visit count, fixation duration, and fixation count as indexes for analysis based on our objectives.

The results of the study suggest that a self-talk intervention allows participants to maintain attention, which in turn promotes motor performance. This is consistent with Hatzigeorgiadis et al. [19] who concluded that people who used a self-talk intervention were effective in improving athletic performance compared to those who did not. According to the strength model of self-control [2], sport performance is affected when participants’ cognitive resources are insufficient. For example, one study found a negative correlation between performance and anxiety especially for participants with temporarily depleted self-control resources [14]. Different from the above findings, the present study found that participants in the self-talk group were still able to achieve better sport performance when self-control resources were extremely scarce, which may be due to the fact that self-talk preserves and enhances self-control, allowing participants to have sufficient cognitive resources to cope with the adverse effects of depletion and distraction interference on athletic performance. Based on Galanis et al.’s [29] conclusion that self-talk counteracts the effects of distraction on performance, the present study further found that participants were able to maintain good motor performance when they were in a state of ego depletion to counteract the auditory distractions, the result that strongly confirms the attentional effects of self-talk when the participants’s cognitive resources were severely limited. Furthermore, consistent with the finding that self-talk can benefit selective attention in participants with ego depletion states [24], the present study concludes that a self-talk intervention can alleviate ego depletion states and benefit attentional focus and motor performance.

The self-talk intervention significantly improved attention functions, according to the results of the post-test eye tracking index. The participants in the self-talk group had significantly longer fixation and visit durations in the near-central and central zones of the dartboard than the participants in the control group. In addition, the self-talk group was more focused on the target zone than the control group, as evidenced by the fixation count in the near-central zone of the dartboard as well as the and visit count and fixation count in the central zone. Our results indicate that self-talk helps minimize distractions when performing tasks [30]. Only the participants who received the self-talk intervention in the ego depletion stage appeared more attentive and more resistant to distracting stimuli during task performance. The findings are also consistent with previous studies [35,36], suggesting that the self-talk intervention enhances attentional control and improves task performance. As described in the previous study, the attention of participants who used self-talk seemed more relaxed and more focused [18,37]. However, compared to the participants in the self-talk group, those in the control group may have found it more difficult to focus their attention under identical experimental conditions. In addition, it is clear from comparing the heat maps and gaze plots for the gaze of the two groups that the participants in the self-talk group paid more attention to the dartboard, particularly the central zone. Consequently, self-talk helped them block out distracting input and stay focused [26,29].

Eye-tracking techniques are attention-guided, a relatively direct and continuous measure of visuospatial attentional processing [46]. Especially in the preparatory stage of motor skills, attention is considered to be one of the most important factors influencing motor performance [47]. By further analysis, the present study found a positive correlation between dart performance and some eye tracking indices in the self-talk group, which is consistent with the findings of previous studies [25,26], which have concluded that self-talk interventions can enhance attention, reduce ego depletion and enhance motor performance. This finding also supports the attentional effect of self-talk through analyzing eye movement behavior to some extent, providing insight into the link between eye movement and motor performance.

It is commonly known that stress is everywhere during sports, pervasive distracting interferences and cognitive resource exhaustion can both hurt motor performance, with a particular emphasis on fine motor sports, which require greater attention. It is possible to conclude that self-talk can improve sports performance by preventing cognitive resource depletion caused by distraction as well as by preserving and renewing attentional resources. Coaches and athletes should consider the insights offered by our study. On the one hand, athletes should insist on using self-talk interventions during practice and competition, as the self-talk intervention can alleviate ego depletion and effectively improve sport performance. In addition, notably, individuals with high levels of self-control are more susceptible to ego depletion because they tend to exert more cognitive effort on attentional focus [48]. So on the other hand, coaches should train their athletes based on the sport and individual traits, choosing the style and content of self-talk that is appropriate for the individual athlete’s traits.

Our study has some limitations; for instance, in the intervention phase, we utilized designated content of instructional self-talk. However, the participants had different personality traits and may have had varying levels of understanding and application of self-talk content. Thus, future research should try to use self-talk content that is suitable for each participant’s personality traits. In addition, the number of participants in the study was small, and future studies could expand the sample size and also consider including dart athletes for a comparative study between experts and novices to explore the moderating role of sport experience in more depth.

While the task-matching hypothesis has been validated [49], additional research is necessary to determine whether motivational self-talk (as opposed to instructional self-talk in the study) is better for performance in fine motor sports when cognitive resources are extremely limited. In addition, sports consume a lot of physical energy, and glucose supplements can improve self-control abilities [50]. Further research should examine the impact of bodily energy level (e.g., physical exhaustion from glucose deprivation) on the effectiveness of self-talk. Moreover, future research should focus on the phenomena of quiet eye and quiet eye training during self-talk. Multidisciplinary techniques — such as physiological, psycho-physiological, and cognitive approaches — can provide more comprehensive knowledge of self-talk [16], and brain science methods such as functional magnetic resonance imaging and electroencephalography may help uncover the neurological underpinnings of self-talk to enhance attention.

## Conclusion

We aimed to determine if self-talk could help participants stay focused and perform better while resisting distractions in a state of ego depletion. The self-talk intervention confirmed that participants were able to improve attention and promote motor performance by using self-talk. At the same time, we used eye-tracking technology to conduct an experiment involving darts and found that compared to the control group, the participants in the self-talk group were more able to improve their attention on the target when cognitive resources were extremely low, thus verifying the attentional effect of self-talk. Future research should consider identify the moderating effect of the sport experience and the neurological mechanism underlying the attentional effect of self-talk. Examining this issue is valuable because it lends theoretical support to the practical application of self-talk as a psychological training tool.

## Supporting information

S1 File Supplementary materials for “The pilot test to verify the validity of the ego depletion transcription material”. (PDF)
