# Supplementary material for: How limited cognitive resources impact the attentional effects of self-talk: An eye-tracking study in dart
Source: PLoS One. 2025 Mar 20;20(3):e0319601. doi: 10.1371/journal.pone.0319601 (PMC11925289; doi:10.1371/journal.pone.0319601)
Supplement: S1 File — (PDF) [file pone.0319601.s001.pdf]

**The content of this file is the pilot test to verify the validity of the ego depletion transcription material.**

# Pilot test

We designed a pilot test to assess the validity of the ego depletion material. We adapted the pilot test from the e-crossing task. Participants in the ego depletion group were required to adjust their copying habits to complete a transcription task within a certain amount of time after being shown a section of text, thereby achieving the impact of ego depletion.

# Method

## Participants

We chose 30 college students (12 male, 18 female), the mean age was 22.07 years (*SD* = 2.63) and randomly assigned them to the ego depletion group (15 participants) and the control group (15 participants). All participants were in good physical and mental health, right-handed, and had either normal or corrected vision. The research protocol was reviewed and approved by the Medical Ethics Committee of the first author's university. The participants provided their written informed consent and their parents agreed to participate in the experiment. The start and end dates of the experiment are September 17, 2023 to October 30, 2023.

## Materials and procedure

Both groups completed a copying task in a quiet, well-lit psychometric lab. A section of Chinese literature was presented to all participants. Those in the ego depletion group were instructed to change their previous writing habits, such as eliminating the "一"

character whenever they came across a Chinese character with a "一" stroke, and to make every effort to replicate the text in the limited time available. Conversely, the control group participants faced no such requirement. Hagger suggested that if the duration of the first task was insufficient, then ego depletion might not occur in the second task [1]. Studies have shown that 8 min has an effective depletion effect [2]. Hence, the transcription task was finally set at 10 min, taking into account the time needed to read the material as well as the time required for preparation.

After both groups performed the transcription task, the Self-Control Manipulation Check questionnaire and the Brief Mood Introspection Scale (BMIS) were administered successively.

## Measures

The Self-Control Manipulation Check questionnaire is a self-report of the difficulty and effort of the transcription task. Answers are rated on a 7-point Likert scale. The following questions are asked: (1) "How difficult did you find it to complete the entire task?" and (2) "How much effort do you think it will take to complete this task?" We selected a revised Chinese version of the BMIS developed by Meyer and Gaschke [3]; it has good reliability and validity, and the internal consistency is 0.948. We measured the internal consistency coefficient, $\alpha = 0.896$, during the pilot test. The scale has 16 items, each of which is rated on a 4-point Likert scale ("completely none," "none," "a little bit," and "completely so"), with items 3, 4, 7, 8, 9, 10, 12, and 15 being reverse scored. After the participants finished the test, each one was asked if he/she had guessed its purpose; they answered that none of them had guessed or were unsure of the test's purpose.

## Data collection and analysis

The pilot test involved a between-subject design. Ego depletion was an independent variable, and the dependent variables were task difficulty scores, effort scores, and overall mood scores. We collected data by filling out questionnaires, and SPSS 22.0 was used for multivariate analysis of variance.

# Results and discussion

The ego depletion task had a significant main effect on task difficulty scores, $F(1,30) = 55.89$, $p = 0.000$, $\eta^2_p = 0.67$, as evidenced by the fact that the participants in the ego depletion group ($M = 5.47$, $SD = 1.06$) scored significantly higher on task difficulty than those in the control group ($M = 2.40$, $SD = 1.18$). A significant main effect of the ego depletion task on effort, $F(1,30) = 21.11$, $p = 0.000$, $\eta^2_p = 0.43$, was demonstrated by the fact that the participants in the ego depletion group ($M = 5.40$, $SD = 1.06$) made significantly more effort than those in the control group ($M = 2.93$, $SD = 1.79$). The ego depletion task had a significant main effect on the total mood scores, $F(1,30) = 6.62$, $p = 0.016$, $\eta^2_p = 0.19$, as evidenced by the fact that the scores of the participants in the ego depletion group ($M = 36.33$, $SD = 7.01$) were significantly lower than the scores of the participants in the control group ($M = 42.13$, $SD = 5.21$).

The participants in the ego depletion group viewed the transcription task's difficulty and effort as considerably higher than those in the control group after altering their writing habits. Alternatively, the control group participants transcribed the literature naturally and did not need to suppress their habitual writing habits; thus, the transcription task was not too difficult for them and did not require much effort. The total mood scores

differed between the two groups, with the scores in the ego depletion group being significantly lower than those in the control group. The participants in the ego depletion group may have found it difficult to break the habit of writing; the continuous transcription task also generated cognitive tiredness in the participants, suggesting a depletion effect of writing transcription material. In sum, the pilot test indicates that the transcription material had the anticipated depletion effect and could be applied to subsequent experiments.

# References

1. Hagger MS, Chatzisarantis NLD, Alberts H, Anggono CO, Batailler C, Birt AR, ... Zwienenberg M. A multilab preregistered replication of the ego-depletion effect. Perspect. Psychol. Sci. 2016; 11(4):546 - 573. https://doi.org/10.1177/1745691616652873 PMID: 27474142

2. Gregersen J, Hatzigeorgiadis A, Galanis E, Comoutos N, Papaioannou A. Countering the Consequences of Ego Depletion: The Effects of Self-Talk on Selective Attention. J Sport Exercise Psy. 2017; 39(3):161-171. https://doi.org/10.1123/jsep.2016-0265 PMID: 28891370

3. Sun CJ. The effect of self-control loss on athletes' performance. Beijing Sport University, 2008.
